# Structural Genomics: Correlation Blocks, Population Structure, and Genome Architecture

**DOI:** 10.2174/138920211794520141

**Published:** 2011-03

**Authors:** Xin-Sheng Hu, Francis C. Yeh, Zhiquan Wang

**Affiliations:** 1 1400 College Plaza, Department of Agricultural, Food and Nutritional Science, University of Alberta, Edmonton, AB T6J 2C8, Canada; 2 Department of Renewable Resources, 751 General Service Building, University of Alberta, Edmonton, Alberta, T6G 2H1, Canada

**Keywords:** Genomic diversity, correlation blocks, multigene family, transposable element, nongenic repeats, GC-rich isochores.

## Abstract

An integration of the pattern of genome-wide inter-site associations with evolutionary forces is important for gaining insights into the genomic evolution in natural or artificial populations. Here, we assess the inter-site correlation blocks and their distributions along chromosomes. A correlation block is broadly termed as the DNA segment within which strong correlations exist between genetic diversities at any two sites. We bring together the population genetic structure and the genomic diversity structure that have been independently built on different scales and synthesize the existing theories and methods for characterizing genomic structure at the population level. We discuss how population structure could shape correlation blocks and their patterns within and between populations. Effects of evolutionary forces (selection, migration, genetic drift, and mutation) on the pattern of genome-wide correlation blocks are discussed. In eukaryote organisms, we briefly discuss the associations between the pattern of correlation blocks and genome assembly features in eukaryote organisms, including the impacts of multigene family, the perturbation of transposable elements, and the repetitive nongenic sequences and GC-rich isochores. Our reviews suggest that the observable pattern of correlation blocks can refine our understanding of the ecological and evolutionary processes underlying the genomic evolution at the population level.

## INTRODUCTION

Determining how much genetic diversity exists in a species and explaining how these diversities coexist in terms of its origin, organization, and maintenance, are of paramount importance in the study of population genetic structure. The analysis of genetic diversity often assumes random recombination of genes at different loci. In such case, the single-locus estimates of genetic diversity and their average across loci are adequate for describing the genetic diversity pattern. However, many selection and non-selective evolutionary forces could cause non-random allelic association among loci. This proposes the necessity to study the joint effects of diversity at multiple loci, i.e. genomic diversity, and the inter-site associations along chromosomes, i.e. the structure of genomic diversity, on the basis of the structured populations.

One approach to assess the structure of population genomic diversity is to measure the association of genetic diversities among linked sites. The DNA segment within which strong (or significant) correlations of genetic diversity exist among linked sites is broadly termed as a correlation block. For instance, the well-known gametic linkage disequilibrium (LD) is the correlation between allele frequencies among sites and the correlation block refers to the haplotype block [1, 2]. Here, the meaning of a correlation block is extended. It can refer to the DNA segment within which the strong correlations exist between heterozygosities (*H_e_* ’s) at linked sites within individual subpopulations, or between population differentiation coefficients (*F_st_* ’s) at linked sites on the same chromosome, or between genetic statistics other than the above variables. Compared with gametic LD, the correlations between *H_e_*’s or between *F_st_*’s among linked sites are higher-order associations. One important difference between the hapolotype block and higher-order correlation block is that we can infer allele linkage phase in the haplotype block. The correlation between *H_e_* ’s or *F_st_* ’s does not require the information on linkage phase. Their commonality is that both correlations suffer from sampling errors. The threshold for determining a block size could vary with the type of correlation block although different blocks might be partially or completely overlapped on the same chromosome [3]. For instance, the logarithm of odds (LOD) is used to determine the square of standardized gametic LD blocks, $r_{D}^{2}$, different from the criteria for determining *D'* blocks [4]. A correlation block itself is a pure statistical concept and its biological meaning is activated only when linked to effects of evolutionary forces. Partial overlapping of different types of blocks on the same chromosomal regions may arise from the effects of distinct evolutionary processes. 

The significance of examining the pattern of correlation blocks (the size, the abundance, and the distribution) is multifold when linked to the effects of evolutionary forces. First, this pattern can gain insights into evolutionary divergences among different chromosomal regions. The chromosomal regions with large block sizes might have experienced evolutionary processes different from the regions with small block sizes, such as the heterogeneity in selection strength, recombination rate, and mutation rate. Second, the pattern may signal regional variation in co-evolution at the population level when positive or negative correlation blocks reveal distinct processes. Third, the pattern can facilitate genetic improvement of quantitative traits when quantitative trait nucleotides (QTN) [5] are mapped within the correlation blocks. The block-based approach is easier to manipulate than the individual single nucleotide polymorphisms (SNPs). 

Current empirical studies on correlation blocks mainly focus on the haplotypic block, such as in the HapMap human genome project [6, 7], and few studies examine other types of blocks and compare these to haplotype blocks. There have been few studies that relate correlation blocks with population genetic structure [8]. The purpose of this synthetic review is to discuss the importance of studying the pattern of correlation blocks in structured populations, complementary to recent reviews on the population genomics where the structure of genomic diversity has not been emphasized [2, 9-11]. Here, we discuss that the pattern of correlation blocks along chromosomes is informative for our inferences on the underlying evolutionary processes. Fig. (**1**) simply illustrates how evolutionary forces could shape the structure of genomic diversity in natural populations. Such structure of genomic diversity could vary with populations and organisms. 

We review the impacts of population genetic structure on the pattern of genome-wide correlation blocks from the theoretical perspective, focusing on the analytical methods that describe this structure and relating the correlation block pattern to evolutionary processes. Previous studies rarely connect conventional population genetic structure with the pattern of genomic diversity, mainly due to the long-term development of two subjects at very different scales and the unavailability of a large number of sequenced genomes. Aspects of genomic evolution that have been evaluated [12] are not considered here, including LD mapping and some statistical issues for outlier detections [13-15]. Our synthesis is different from previous reviews on genomic structure from a variety of perspectives [16, 17]. Here, we discuss the pattern of correlation blocks within and between populations. We then deliberate on the possible relations between the pattern of correlation blocks and the genome architecture in eukaryotic organisms, including the effects of multigene families, transposable elements (TE), and nongenic sequences and GC isochores.

## CORRELATION BLOCKS WITHIN POPULATIONS

### Mechanisms for Maintaining Inter-Site Correlations

Variables for calculating the inter-site correlation blocks may refer to those that denote genetic variation within populations, such as allele frequency and heterozygosity. The biological significance for the inter-site correlations of these variables can be activated only when they are associated with the evolutionary forces. Mechanisms for maintaining inter-site correlations are complicated from the evolutionary perspectives. For a given pair of linked SNP sites, the correlation can be one of three combination types: selective-by-selective, selective-by-neutral, and neutral-by-neutral sites. Correlation between linked selective sites can result from a variety of selection systems. The interaction for the selective-by-selective combination depends on the type of selection system at individual sites (e.g., directional, heterozygous advantage/disadvantage, antagonistic, and frequency-dependent selection). As the number of combinations increases, it becomes progressively difficult to reveal the relative contributions of differently combined selection systems. For instance, the distinction becomes difficult even for different types of balancing selection [12, 18]. The correlation between selective sites can be enhanced in structured populations where immigration facilitates their LDs [19, 20]. Heterogeneity in selection systems in different regions on the same chromosomes facilitates different extents of inter-site correlations.

Correlation between linked selective and neutral sites is also complex, especially when multiple selective sites jointly change the linked neutral sites [21]. The indirect effects come either from the background selection owing to the deleterious mutation at the selective sites [22] or from the hitchhiking effects owing to the advantageous mutation at the selective sites [23]. The transient correlation between selective and neutral sites can be reinforced where immigration is present, as implied from the results in the cytonuclear system [21]. Heterogeneity in background selection or in genetic hitchhiking effects in different regions of the same chromosome enhances transient blocks with varying sizes.

Correlations between linked neutral sites are often transient due to the effects of recombination and are related to the number and the length of neutral DNA sequence segments. The transient correlation between neutral sites can arise from genetic drift for the populations with a short history [24] and/or from the effects of immigration. Introns with various secondary structures (e.g., Groups I and II introns) involve tight linkage between distant sites. The chromosomal regions with the consecutive neutral sites, such as some noncoding or intron DNA sequences regions, eventually form the intervals that flank different correlation blocks. For instance, the average length of introns in human genomes is 4.66kb which generates an enormous number of tiny islands of exons with an average length of 0.15kb [17, p.49]. This implies that on average, the block sizes are probably smaller in the human genomes than in species with smaller sizes of introns, such as in *Caenorhabditis* and *Drosophila* [17, pp. 49-50]. 

Statistically, a significant gametic LD is the basis for maintaining inter-site correlations since higher-order inter-site associations are the function of lower-order associations [2, 13, 25-27]. The distribution pattern of gametic LD along chromosomes is associated with the heterogeneous recombination rates [28-31] which generate the inter-site correlation blocks of different sizes along the chromosomes. Evolutionary mechanisms for maintaining gametic LD can directly or indirectly affect higher-order inter-site associations although the reverse relationships are not true. Higher-order inter-site correlations can arise from the interactions other than gametic LD, such as zygotic epistasis between linked sites. There is no an one-to-one corresponding relationship in mechanism between lower- and higher-order LDs. 

### Methods for Measuring Correlation Blocks

Biologically, mapping correlation blocks is different from mapping genetic variation at individual sites since the former reflects the inter-site associations while the latter does not. For instance, an IBD (identity by descent) map describes the diversity at individual sites and cannot tell the co-evolution process between the linked sites [32, 33]. Correlation block maps can reveal the pattern of co-evolutionary variations along the chromosomes. For instance, methods for estimating the correlations of pairwise relatedness coefficients at linked sites [33] and for estimating the correlations of non-allele descents [34] can be applied to constructing the inter-site association maps. Other methods, such as the wavelet analysis [29] and the joint estimates of multilocus inbreeding coefficients [35, 36], can also be used.

One common measure of inter-site association is the square of standardized gametic LD, $r_{D}^{2}$, that describes the correlation of allele frequencies between linked sites [28,37]. This statistics is different from the correlation of pair-wise relatedness or the correlation of heterozygosities, given that different components of the genetic variation are used [38]. Information on either inter-site IBD or within-site IBD is not singled out in the gametic LD or $r_{D}^{2}$ mapping. Only information on the identity in state (IIS) is in use, even when IIS is the function of IBD [33, 34, 39]. Their resulting maps for correlation blocks along chromosomes are different due to their different sensitiveness to the effects of recombination that reduces the probability of inter-site IBD for a given pair of linked nuclear sites. The correlation block map for non-allele- or allele- descent measures can be different from the gametic LD or $r_{D}^{2}$ blocks in signaling the co-evolution process due to natural/artificial factors. This can occur in the large population with a long history where only small descent blocks survive, contrast to the population with a short history where large descent correlation blocks exist. 

Correlation of heterozygosities describes an alternative pattern of genomic diversity although zygotic LD is a complicated function of gametic LD [25, 27]. To examine their differences, we synthesize the existing theories to calculate the correlation of heterozygosities in a solely neutral process. Consider two diallelic neutral SNP sites with the recombination rate *r* in a random mating population of effective size *N_e_*. Let *A*_1_ and *A*_2_ be the alleles at site *A*, with the initial allele frequencies *p_A_* and *q_A_*, respectively; *B*_1_ and *B*_2_ be the alleles at site *B*, with the initial allele frequencies *p_B_* and *q_B_*, respectively. Let *H_A_* and *H_B_* be the heterozygosities at sites *A* and *B*, respectively. The correlation coefficient of heterozygosities at generation *t*, *R_t_*, is calculated by

(1) $$R_{t}=\mathrm{cov}\left(H_{A},H_{B}\right)/\left(\sigma_{H_{A}}\sigma_{H_{B}}\right),$$

where cov(*H_A_*,*H_B_*) = *E*(*H_A_H_B_*) - *E*(*H_A_*)*E*(*H_B_*), in which *E*(*H_A_*), *E*(*H_B_*), and *E*(*H_A_H_B_*) are the expectations of heterozygosity at *A*, *B*, and both sites, respectively; $\sigma_{H_{A}}^{2}$ and $\sigma_{H_{B}}^{2}$ are the variances of heterozygosity at sites *A* and *B*, respectively.

The expected heterozygosity at site *A* is $E\left(H_{A}\right)=2p_{A}q_{A}\gamma_{1}^{t}$( *p_A_* and *q_A_* are equal to the averages of allele frequencies over all possible outcomes caused by genetic drift effects), and $\gamma_{1}=1-1/{2N}_{e}.E\left(H_{B}\right)$ can be expressed in a similar way. The variance of heterozygosity at site *A*, $\sigma_{H_{A}}^{2}$, is calculated by $E{\left(H_{A}\right)}^{2}-\left({E\left(H_{A}\right)}^{2}\right)$. Using the formulae derived by Robertson [40, pp. 203-206], we can obtain

(2) $$\sigma_{H_{A}}^{2}=4p_{A}q_{A}\left(\frac{1}{5}{\mathrm{-p}}_{A}q_{A}\gamma_{1}^{t}\right)\gamma_{1}^{t}-4p_{A}q_{A}\left(\frac{1}{5}-p_{A}q_{A}\right)\gamma_{2}^{t},$$

where $\gamma_{2}=\gamma_{1}\left(1-1/N_{e}\right)\left(1-3/2N_{e}\right).\sigma_{H_{B}}^{2}$ can be readily obtained by replacing subscript *A* in the above equation with subscript *B*. 

Following Ohta and Kimura [41, p. 52], the expected frequency of double heterozygotes at generation *t* is

(3) $$E\left(H_{A}H_{B}\right)=4\sum_{i=1}^{3}C_{\mathrm{Hi}}\left(\frac{p_{A}q_{A}p_{B}q_{B}}{2\left(1+\lambda_{i}\right)}+\frac{1}{4}\left(3+4N_{e}r+2\lambda_{i}\right)D_{0}\left(1-2p_{A}\right)\left(1-2p_{B}\right)+D_{0}^{2}\right)\exp\left(\lambda_{i}t/N_{e}\right),$$

where *λ_i_* is the constant related to the decaying rate of , *E*(*H_A_H_B_*), *C_Hi_* is the function of *λ_i_* [41, p.52], and *D*_0_ is the initial linkage disequilibrium in the population. Fig. (**2A**) shows how *R_t_* changes with the time and with the recombination rate, indicating that the strong transient correlation blocks are present only within a short distance (tightly linked sites). Fig. (**2B**) shows that the transient gametic LD, $D_{t}=\exp\left(-\left({2N}_{e}r+1\right)t/\left(2\mathrm{Ne}\right)\right)D_{0}$ [42], decays faster with time within short distances than the transient zygotic LD, cov(*H_A_*, *H_B_*), although gametic LD is greater than zygotic LD within a short range. The presence of natural or artificial selection may lead to the pattern biased from the expectations in a pure neutral process. This remains to be explored in theory.

As an example, we compared the structures of zygotic and gametic LDs on one human chromosome (Chr.21) from CHB-Han Chinese Beijing population. Data were downloaded from ftp://ftp.sanger.ac.uk/pub/hapma3/r3 from the Human Genome Project group at the Wellcome Trust Sanger Institute. There were 137 individuals in this population and 18707 SNPs on Chr. 21 (the chromosome with the smallest number of SNPs in this population). After removing those SNPs with minor allele frequency (MAF) smaller than 0.05, 15817 SNPs were used for both zygotic and gametic LDs analyses. Fig. (**3A**) shows the pattern of pairwise gametic and zygotic LDs with the distance, evidencing that the correlation of heterozygosity was generally weaker than gametic LD. A significant difference existed between the distribution of correlation of heterozygosity and the distribution of gametic LD (Fig. **3B**; Kolmogorov-Smirnov test, p-value <2.2×10^-16^). Fig. (**4A**) shows that the distributions of SNP markers each with at least strong gametic ($r_{D}^{2}>0.3$ ; green color lines) or zygotic ($r_{H}^{2}>0.3$ ; red color lines) associations with its neighbor markers. Some chromosomal regions formed correlation blocks with different sizes. Different blocks between gametic and zygotic associations can be visualized (the exact data not shown here). 1292 of the 15817 SNPs (8.2%) showed strong gametic LDs ($r_{D}^{2}>0.3$) but weak heterozygosity correlations, but only 11 of the 15817 SNPs (0.07%) showed the reverse pattern (Fig. **4B**). However, when highly strong gametic and zygotic associations are considered, say $r_{D}^{2}>0.9$ and $r_{H}^{2}>0.9$, the same pattern of correlations was observed between them (data not shown here for Chr. 21 in CHB population). Only those tightly linked sites maintained strong gametic and zygotic LDs. Our analysis of human chromosome 21 clearly shows that gametic and zygotic LDs had distinct structures of genomic diversity. A further analysis is of interest to map the functional meanings of these distinct SNPs in gene expressions. 

Theories previously used to measure the inter-site structure at the sequence level are useful to describe genomic structure at the population level, such as in auto-correlation and spectral analysis [43-45]. The difference is that the variables here refer to the genetic diversities at individual sites other than the nucleotide compositions. These will likely produce different patterns of genomic diversity along the chromosomes, and some of them are probably not related to the haplotypic LD block pattern.

### Density Distribution of Correlation Blocks

One way to summarize the pattern of correlation blocks is to look at the density distribution of correlation block sizes, similar to the method for describing the distribution of nucleotide base composition at the sequence level [43]. This can give a general picture about inter-site associations on a chromosome. The sizes of correlation blocks could be altered under the effects of evolutionary forces. Whether the density distribution of block sizes is a stable or not remains to be studied in theory under the balancing effects of recombination and other evolutionary forces.

Fig. (**5A**) shows the abundance distribution of strong pairwise gametic and zygotic LDs ($r_{D}^{2}>0.3$ and $r_{H}^{2}>0.3$) on the human Chr.21 from the CHB population. This is a negative exponential distribution, with a large number of pairwise correlations within short distances and a small number of correlations within large distances. Fig. (**5B**) displays the density distribution of gametic LD block sizes, measured in terms of Lewontin’s *D'* [46], which shows a kind of negative exponential distribution. This is probably related to the long-time history of human population where the effects of recombination were substantial, leading to a majority of small gametic LD blocks. Distribution other than the negative exponential kind cannot be excluded under the impacts of evolutionary forces, such as the non-exponential distribution of gametic LD block sizes in domestic dairy and beef cattle populations caused by long-term directional artificial selection (Li *et al*., unpublished data).

### Perspectives

The outstanding challenge is how to unravel the relative effects of evolutionary forces (mutation, migration, selection, and drift) in forming the pattern of correlation blocks, given the observed block sizes and their distribution pattern. If we examine the average correlation block size and its variation (e.g., its standard deviation) at the genome-wide scale, these evolutionary forces can produce distinct patterns. Natural selection and mutation can, on average, bring about smaller correlation blocks and a higher variation in block size in a large population with a long history than in a small population with a short history. This is because selection and mutation can cause regional genetic variation along the chromosomes, and a long-time history facilitates the collapse of LD due to the effects of recombination. 

**Fig. (1):**
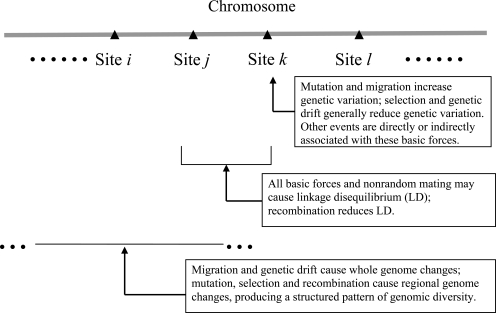
This diagram illustrates the effects of basic evolutionary forces (selection, mutation, migration, and genetic drift) on genomic diversity in natural populations. The pattern of genomic diversity along chromosomes can be assessed when multiple sites and their linkage phases along the chromosomes are assessed simultaneously, which turns the conventional population genetics studies into a large genome scale.

**Fig. (3):**
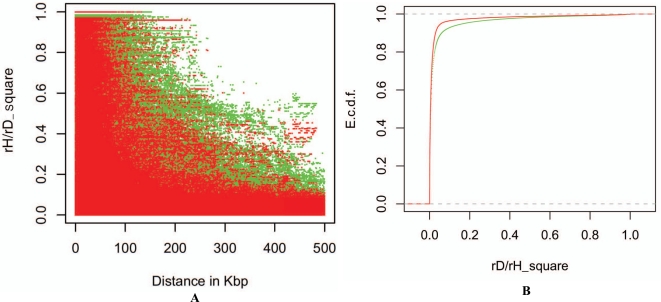
A. Distribution of pairwise correlations of heterozygosities (red dots), $r_{H}^{2}$ , and gametic LD (green dots), $r_{D}^{2}$ , with distance on human chromosome 21 in CHB (Han Chinese Beijing) population, indicating that $r_{H}^{2}$ collapsed faster than $r_{D}^{2}$ with distance. **B**. Patterns for the empirical cumulative distribution function (e.c.d.f.; green for $r_{D}^{2}$ ’s and red for $r_{H}^{2}$ ’s ). Kolmogorov-Smirnov test indicated that there was a significant difference between $r_{H}^{2}$ and $r_{D}^{2}$ distributions, with p-value < 2.2×10^-16^.

**Fig. (4):**
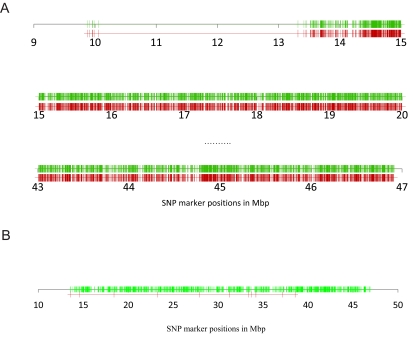
**A**. Distribution of SNP markers each with at least one pairwise $r_{D}^{2}$ > 0.3 (green color lines) or $r_{H}^{2}$ > 0.3 (red color lines) on human chromosome 21 in CHB (Han Chinese Beijing) population, evidencing many distinct chromosomal regions between gametic and zygotic associations. **B**. The green color lines represented the positions of SNP markers that were present in the subset of SNPs with strong paiwise gametic LDs ( $r_{D}^{2}$ >0.3) but absent in the subset of SNPs with strong zygotic LDs ( $r_{H}^{2}$ >0.3); the red color lines for the reverse case results.

Genetic drift and immigration can increase the average correlation block size for the population with a short history. For the population with a long history, however, the size of correlation block produced by genetic drift decays with time, and this, on average, results in smaller block sizes. The block sizes and their distribution donot reveal immigration effects for a single subpopulation because immigration changes the whole genome-wide LD (Fig. **1**). To infer the effects of immigration and genetic drift, a comparison among populations is necessary in terms of average correlation block size and distribution variances. The block sizes and their distributions vary with the populations of various demographic histories, as implied from the comparisons of LD blocks in different soybean populations [4]. The expected correlation block is greater for a small population with a short history than with a long history due to the collapse of LD by recombination rate and the effect of genetic drift with time. However, this is likely not the case for the effects of immigration whose effects can increase the average size of correlation block.

## CORRELATION BLOCKS AMONG POPULATIONS

Variables for calculating the inter-site correlations among populations may be Wright’s *F_st_* or other genetic statistics (e.g., Nei’s genetic distances at individual sites [47]). The chromosomal regions with smaller *F_st_* ’s and larger *F_st_* ’s at linked sites imply their more convergent and divergent evolution among populations, respectively. Each of these two regions may possess positive inter-site *F_st_*-correlations. The *F_st_*-correlation block is hitherto not assessed despite *F_st_* maps are available in human, cattle, and other organisms [48, 49]. 

Investigating the inter-site *F_st_* correlations is different from investigating the inter-site correlations within populations. First, a strong positive or negative *F_st_*-correlation indicates that the linked sites undergone similar or different evolution processes in different populations, respectively. Heterogeneous variation in *F_st_*-correlation along chromosomes indicates the presence of different effects of evolutionary forces. Second, patterns of *F_st_*-correlation blocks are informative on genetic conservation at the population level since genetic variation within blocks provides redundant information among populations. This aids the block-based approach to be more effective in utilizing genome-wide divergences among populations in conservation.

### Mechanisms for Maintaining Inter-Site *F_st_* -Correlations

In principle, the process that increases the inter-site LDs within populations and the allele frequency differentiation among populations at individual sites can facilitate inter-site *F_st_* correlations. Statistically, *F_st_*-correlation is related to gametic LD within and between populations. The processes are very complicated when linked to the effects of evolutionary forces. For a pair of linked selective-by-selective sites, synergistic interactions enhance inter-site *F_st_*-correlations while antagonistic interactions reduce *F_st_*-correlations. Different forms of selection create a potentially large number of selection-by-selection combinations. One speculation is that differential selection among the populations reduces the average *F_st_*-correlation block size. For instance, selection intensities at given sites in the central populations are different from those in the marginal populations. Consequently, this changes the distribution pattern of *F_st_*-correlation blocks between the central and marginal populations. For a pair of linked selective-by-neutral sites, genetic hitchhiking and/or selective sweep effects increase transient *F_st_*-correlations. This case becomes even more complex when multiple selective sites are involved in changing a commonly linked neutral site [50, 51].

For a pair of linked neutral-by-neutral sites, genetic drift and migration help to maintain transient *F_st_*-correlations but they are different in process. Although genetic drift can bring about the whole genome changes, the difference in effective population size among populations can reduce the *F_st_*-correlation block sizes on average and change their distribution along chromosomes. This can be implied from the empirical observations of small LD blocks in the derived populations owing to the different demographic histories, such as the founder effects [52]. The transient LD initially generated by genetic drift gradually decays with time owing to recombination [42]. This is also the same case for the change of transient *F_st_* correlations for a pair of linked neutral sites. Unlike the effects of genetic drift, LD generated by migration could be maintained as long as the inter-population migration takes place [19, 50]. Thus, on average, a large *F_st_*-correlation block might increase although *F_st_* ’s at individual sites decrease as the migration rate increases [53].

Similar to the effects of migration, neutral mutation reduces population differentiation (e.g., $F_{\mathrm{st}}={\left(1+{2N}_{e\left(\mathrm{local}\right)}\left(\tilde{m}+v\right)\right)}^{-1}$ under the classical infinite island model, *v* is the mutation rate [53]). This facilitates genomic convergence among populations and increases the *F_st_*-correlation block sizes. However, this may not be the case for selective sites where mutants favorable to different habitats increase *F_st_* [54] and produce different associations with linked sites on the same chromosomes. The joint effects of mutation and selection can increase or decrease the *F_st_*-correlation block sizes, depending upon whether the joint effects are consistent across subpopulations or not.

Again, the remaining challenge is to disentangle the relative effects of different evolutionary forces from the pattern of *F_st_*-correlations. Migration and genetic drift help to increase the average size of *F_st_*-correlation block but selection and mutation facilitate to produce the pattern of various block sizes. These results vary with the structure and history of populations.

### Methods for Measuring Inter-Site *F_st_* -Correlations

Several software packages are currently available to estimate *F_st_* at individual sites, but estimation of *F_st_*-correlation has not been fully developed [25]. Here, we discuss the application of the method developed by Cockerham and Weir [55] for estimating *F_st_*-correlation. Consider population genomic datasets where all sampled individuals are sequenced as in genomes of human and cattle populations that are publically available. Pairs of alleles at each of two linked sites fall into two genic hierarchical levels: alleles in different individuals in the same subpopulation and alleles in different subpopulations in the same population. Let *x_ikl_* be the indicator variable, where *i* indicates the location of the allele, *k* and *l* are the alleles at the first and second site, respectively. When the alleles are *k* and *l* at the first and second sites, respectively, *x_ikl_* = 1; which otherwise equals zero, xik'l' = 0k'≠k, or l'≠l,or both . The expectation of *x_ikl_* across all subpopulations is *E*(*x_ikl_*) = *p_kl_* where *p_kl_* is the gametic frequency. The variance of this indicator variable follows a binomial distribution, $E\left(x_{\mathrm{ikl}}^{2}\right)-{\left(E\left(x_{\mathrm{ikl}}\right)\right)}^{2}=p_{\mathrm{kl}}\left(1-p_{\mathrm{kl}}\right)$. 

Let $\theta_{{\mathrm{ii}}^{'}\left(\mathrm{kl}\right)}$ be the correlation between *x_ikl_* and *x_i'kl_*, *θ_ii'(k)_* be the correlation between *x_ik_* and *x_i'k_* at the first site, and *θ_ii'(l)_* be the correlation between *x_il_* and *x_i'l_* at the second site. *θ_ii'(k)_* and *θ_ii'(l)_* can be estimated using the analysis of variances (ANOVA). Using the same notation as Cockerham and Weir [55], let *θ_n_* = *θ_ii'_* where *n* is set as 1 when *i* and *i'* are from the same subpopulation (*i* = *i'*), and *n*=2 when *i* and *i'* are from different subpopulations. The expectation of a pair of alleles each from different sites can be expressed as

(4) Exiklxi'kl = pkl2+θii'klpkl1−pkl.

The correlation at two sites *θ_ii'(kl)_* can be further decomposed as

(5) θii'kl = θii'kθii'l+covθii'k,θii'l.

The *F_st_*-correlation can be calculated by $\mathrm{cov}\left(\theta_{1\left(k\right)},\theta_{1\left(l\right)}\right)/{\left(\mathrm{var}\left(\theta_{1\left(k\right)}\right)\mathrm{var}\left(\theta_{1\left(l\right)}\right)\right)}^{1/2}$ where var(*θ*_1(*k*)_) and var(*θ*_1(*l*)_) can be estimated using conventional methods, such as bootstrapping.

To employ Cockerham and Weir’s [55] method for estimating $\theta_{1\left(\mathrm{kl}\right)},Q_{n}=\sum_{k}\sum_{l}E\left(x_{\mathrm{ikl}}x_{i'\mathrm{kl}}\right)$ can be expressed as

(6) $$Q_{n}=q+\theta_{n\left(\mathrm{kl}\right)}\left(1-q\right),$$

where $q=\sum_{k}\sum_{l}p_{\mathrm{kl}}^{2}$. Here the correlation *θ_n(kl)_* is a constant for the two given sites. Eq. (6) has the same form as Cockerham and Weir ([55], p.8512). Only two-level hierarchy components are considered: variance within subpopulations ($\sigma_{1}^{2}$) and variance among subpopulations ($\sigma_{2}^{2}$), where $\sigma_{1}^{2}=1-Q_{1}=\left(1-\theta_{1\left(\mathrm{kl}\right)}\right)\left(1-q\right),\sigma_{2}^{2}=\theta_{1\left(\mathrm{kl}\right)}\left(1-q\right)$ and $\theta_{1\left(\mathrm{kl}\right)}=\sigma_{2}^{2}/\left(\sigma_{1}^{2}+\sigma_{2}^{2}\right).$ Once *θ_1(kl)_* is available using ANOVA [25, pp. 171-176], cov(*θ_1(k)_*,*θ_1(l)_*) can be estimated from Eq. (5).

Since *F_st_* calculation is related to heterozygosities in the subpopulations and global population, *F_st_*-correlation is related to the correlation of heterozygosities at the two levels. Wright [53] showed that 1 - *F_it_* = (1-*F_st_*)(1-*F_is_*) from which we can show that the *F_st_*-correlation is related to the correlation of heterozygosities at the global (*H_	it_*) and local (*H_	is_*) levels. For a two linked sites (*i* and *j*), we can obtain

(7) $$\mathrm{cov}\left(H_{{\mathrm{it}}_{i}},H_{{\mathrm{it}}_{j}}\right)=\mathrm{cov}\left(H_{{\mathrm{is}}_{i}},H_{{\mathrm{is}}_{j}}\right)+\mathrm{cov}\left(F_{{\mathrm{st}}_{i}},F_{{\mathrm{st}}_{j}}\right)-\Delta ,$$

where

$\Delta =\mathrm{cov}\left(H_{{\mathrm{is}}_{i}},F_{{\mathrm{st}}_{j}}H_{{\mathrm{is}}_{j}}\right)+\mathrm{cov}\left(H_{{\mathrm{is}}_{j}},F_{{\mathrm{st}}_{i}}H_{{\mathrm{is}}_{i}}\right)+\mathrm{cov}\left(F_{{\mathrm{st}}_{i}},F_{{\mathrm{st}}_{j}}F_{{\mathrm{is}}_{j}}\right)+\mathrm{cov}\left(F_{{\mathrm{st}}_{j}},F_{{\mathrm{st}}_{i}}F_{{\mathrm{is}}_{i}}\right)-\mathrm{cov}\left(F_{{\mathrm{st}}_{i}}F_{{\mathrm{is}}_{i}},F_{{\mathrm{st}}_{j}}F_{{\mathrm{is}}_{j}}\right)$.

 The inter-site *F_st_* co-variance is related to the inter-site co-variance of heterozygosities at the global and local levels. This also implies that the inter-site *F_st_* co-variance is ultimately associated with the gametic LD at the global and local levels. 

### Local and Global Gametic LDs

The difference between inter-site heterozygosity correlations at the global and local levels is related to inter-site *F_st_*-correlation. If population differentiation is absent, inter-site correlation of heterozygosities should be equal at the two levels. Thus, the inter-site *F_st_*-correlation can be perceived from the change of glocal and local gametic LDs since zygotic associations are the function of gametic LD [27]. Here, we briefly discuss the global and local LDs in structured populations that indirectly affect the *F_st_* correlation and its distribution.

The amounts of global and local LDs are different due to unequal rates of decay. This facilitates the divergence between the pattern of correlation blocks within the whole population (e.g., the pattern of LD blocks or *H_e_* correlation blocks) and the pattern of *F_st_*-correlation blocks among sub-populations. For instance, we may compare the collapse of two transient LDs by synthesizing the results of Wright [56] and Hill and Robertson [42] in a purely neutral process. Suppose that a population is subdivided into *n* sub-populations each with the same constant effective size *N*_e(*local*)_. Random sampling acts independently on individual subpopulations. Consider two diallelic linked neutral sites with the recombination rate *r* between them. Assume that all subpopulations begin from the same allele frequencies as in the entire population. Let *D*_0_ be the initial gametic linkage disequilibrium in the global population or in any initial sub-population. According to Wright [53], population differentiation *F*_*st*(*t*)_ at each neutral site at generation *t* can be expressed as

(8) $$F_{\mathrm{st}\left(t\right)}=1-{\left(1-\frac{1}{{2N}_{e\left(\mathrm{local}\right)}}\right)}^{t}$$

From Hill and Robertson [42], the expected LD in each subpopulation at generation *t*, *E*(*D*_local(*t*)_), is expressed as

(9) $$E\left(D_{\mathrm{local}\left(t\right)}\right)=\left(1-r\right)\left(1-\frac{1}{{2N}_{e\left(\mathrm{local}\right)}}\right)E\left(D_{\mathrm{local}\left(t-1\right)}\right)={\left(1-r\right)}^{t}{\left(1-\frac{1}{{2N}_{e\left(\mathrm{local}\right)}}\right)}^{t}D_{0}.$$

Let $N_{e\left(\mathrm{global}\right)\left(t\right)}$ be the effective global population size at generation *t*. From Wright [56], $N_{e\left(\mathrm{global}\right)\left(t\right)}$ can be expressed as

(10) $$N_{e\left(\mathrm{global}\right)\left(t\right)}=\frac{{\mathrm{nN}}_{e\left(\mathrm{local}\right)}}{{1-F}_{\mathrm{st}\left(t\right)}}={\mathrm{nN}}_{e\left(\mathrm{local}\right)}{\left(1-\frac{1}{{2N}_{e\left(\mathrm{local}\right)}}\right)}^{-t}$$

Let *D*_global(t)_ be the expected global LD. From Hill and Robertson’s [42] and Eq. (10), we obtained *E*(*D*_global(*t*)_):

(11) $$\begin{array}{c} E\left(D_{\mathrm{global}\left(t\right)}\right)={\left(1-r\right)}^{t}\prod_{i=0}^{t-1}\left(1-\frac{1}{{2\mathrm{nN}}_{e\left(\mathrm{local}\right)}}{\left(1-\frac{1}{{2N}_{e\left(\mathrm{local}\right)}}\right)}^{i}\right).\\ \approx {\left(1-r\right)}^{t}\left(1-\frac{1}{n}\left(1-{\left(1-\frac{1}{{2N}_{e\left(\mathrm{local}\right)}}\right)}^{t}\right)\right). \end{array}$$

Combining (8), (9), and (11) yields

(12) $$\frac{E\left(D_{\mathrm{global}\left(t\right)}\right)}{E\left(D_{\mathrm{local}\left(t\right)}\right)}=\frac{{1-F}_{\mathrm{st}\left(t\right)}/n}{{1-F}_{\mathrm{st}\left(t\right)}}.$$

Fig. (**6**) shows that the local LD reduces more rapidly with time than the global LD as *F_st_* increases in a purely neutral process. This is because population differentiation increases the effective global population size in a pure neutral process, which in turn reduces the genetic drift and enhances the global LD.

In the presence of other evolutionary forces, such as the change of local LD by the joint effects of interpopulation gene flow and natural selection [50], the relationships could be biased from the expectation under the neutral process. The relationship between *N*_*e*(global)_ and *N*_*e*(local)_ becomes more complex in the presence of natural selection: $N_{e\left(\mathrm{global}\right)}={\mathrm{nN}}_{e\left(\mathrm{local}\right)}{\left(\left(1+V\right)\left(1-F_{\mathrm{st}}\right)+2N_{e\left(\mathrm{local}\right)}F_{\mathrm{st}}V\right)}^{-1}$ (*V* is the variance in fitness among subpopulations) for the selective sites [57]. Also, population differentiation for plant species becomes $F_{\mathrm{st}}={\left(1+2N_{e\left(\mathrm{local}\right)}\tilde{m}n^{2}/{\left(n-1\right)}^{2}\right)}^{-1}$ (*n* is the number of subpopulations; the migration rate $\tilde{m}$ has different forms for alleles with different modes of inheritance in plants) for neutral sites [58, 59]. All these scenarios can change the global LD. The global genetic drift for the joint neutral sites is not the same as that for the joint selective sites. Similarly, the global LD affecting the joint neutral sites is not the same as that affecting the joint selective sites. An intermediate situation is the transient global LDs between the selective and neutral sites since genetic hitchhiking modifies their LDs and the LDs in local populations. These different scenarios can affect *F_st_* correlation blocks and their distribution along the chromosomes.

### Density Distribution of *F_st_*-Correlations 

There are few empirical studies on the density distribution of *F_st_*-correlation blocks. Some reports are available about the density distribution for individual *F_st_* ’s [60]. In a neutral process, genetic drift and recombination gradually erode *F_st_*-correlations while migration increases *F_st_*-correlation. This eventually leads to a steady-state distribution in *F_st_* [53] and *F_st_*-correlation. The non-random distribution of recombination along chromosomes facilitates the generation of different *F_st_*-correlation blocks. A shorter distance has correspondingly, a lower recombination rate and helps to maintain smaller haplotypic blocks. Compared with the gametic LD, *F_st_*-correlation (higher-order) is also weaker. It is contemplated that there are a larger number of small *F_st_*-correlation blocks and a few large blocks, displaying a highly skew distribution. 

Selection can modify the distribution of *F_st_*-correlation blocks. If one block contains only one selective site (e.g., adaptive QTN) together with many neutral sites, the distribution of the sizes of *F_st_* correlation blocks on the whole reflects the distribution of the effects of selective sites along the chromosomes. The site with a large selective intensity or gene effect is expected to have a large size of *F_st_* correlation block due to the effects of genetic hitchhiking. The number of blocks is likely equal to the number of *F_st_* -outliers [54]. If the effects of all selective sites follow a gamma distribution [61, 62], it is hypothesized that the size of *F_st_* correlation block may likely follow the same kind of distribution. When multiple selective sites are involved in the individual *F_st_* correlation blocks, the number of *F_st_* correlation blocks is unequal to the number of selective sites. The distribution of block size likely exhibits the type other than the negative exponential distribution. This requires further empirical tests.

When other genetic statistics, such as Nei’s distance, are used to describe the population genetic differentiation, different block sizes and distribution patterns could be produced on the same chromosome. The sensitivity to population differentiation at individual sites has not been compared among *F_st_* and other statistics. Differential sensitivities to natural selection and genetic hitchhiking effects can influence the sizes of correlation block and their distribution patterns for a given array of subpopulations. 

## GENOME ARCHITECTURE AND CORRELATION BLOCKS

Eukaryotic genome assembly has some explicit features, such as the presence of multigene families and transposable elements (TE). These features could affect the size and distribution of correlation blocks within and between populations. Here, we separately discuss these potential effects, including the effects of multigene families, TE, and sequence repeats. In each case, we begin by discussing the effects of these features on the correlation blocks within population (gametic or zygotic associations), followed by their effects on *F_st_* -correlation blocks among populations. 

### Effects of Multigene Families

Multigene families account for some percentages of the whole genome. Consider the correlation blocks within sub-populations in terms of gene family. Empirical studies on the relation between inter-site associations and multigene families are not available, but the density distribution of multigene family size has been reviewed in model organisms [17]. One conjecture is that multigene families could shape the correlation blocks and their distribution in two ways or in their mixture. One is that each family member can form one or more correlation blocks. The other is that partial segments of each family member are involved in the correlation blocks. 

Correlation blocks can be altered by the processes that generate and maintain multigene families. Gene conversion and unequal crossing-over are the common processes although others for concerted evolution have been proposed [63, 64]. A biased gene conversion driven by natural selection can accelerate the homogeneity among the family members. When the evolution of multigene families is in the steady state, individual correlation blocks in terms of family member are likely similar in size even if the number of members varies among the individuals. When the evolution of multigene families remains in a transient state, a variant repeat does not completely spread to all other family members, and the sizes of correlation blocks could vary substantially among the family members. Similar outcomes can be expected when the multigene families change through unequal crossing-over. Theoretical studies have shown that the probability of identical multigene family members exponentially decreases with their distance along the chromosomes [65], implying the presence of correlated blocks among family members under the neutral hypothesis (mutation, genetic drift, intrachromosomal unequal crossing-over, and interchromosomal equal crossing-over).

The sizes of correlation blocks are related to the structure and function of the multigene family and the interspersed coding/non-coding sequences between family members. The number and lengths of noncoding regions within each family member affect the genetic divergence among members owing to the different mutation rates between coding and non-coding regions. Consequently, this acts as a biological barrier to the spread of advantageous variants to all other members through unequal crossing-over and modifies the distribution of correlation blocks. When unequal selection intensities exist among the interspersed segments, the size of correlation block in terms of family member should change. When the interspersed sequences are the solely noncoding sequences, an explicit separation between the individual blocks is expected.

With a reference to the *F_st_*-correlation blocks in terms of family member, distinct selection facilitates gene conversion or unequal crossing-over. However, the spread of locally adaptive variants to other members might not be at the same speed among populations. As a result, the sizes of *F_st_* -correlation blocks may vary with the family members. 

The exchange of genomes among populations acts as a biological barrier to the spread of locally adaptive variants among family members when variants in the migrating genomes are maladaptive to the recipient populations, similar to the presence of migration loads-the reduction of population fitness due to maladaptive immigrants [66, 67]. Recombination of immigrated maladaptive variants with resident genomes *via *a certain mating system reduces the mean fitness in recipient populations. However, genome replacement of the local populations can be accelerated when all the members or the majority of members of the multigene family in the migrating genomes are more adapted to the local populations ([53], pp.36-38). The spread of adaptive variants to all other members can increase when the rate of gene conversion or the rate of unequal crossing-over is high. The *F_st_*-correlation blocks and their distribution in terms of multigene family members quickly converge among populations, analogous to the function of gene flow in reducing population differentiation at a single locus. 

**Fig. (7):**
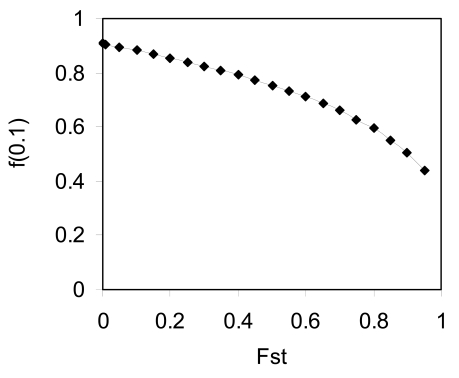
Effects of population differentiation on the identity coefficient between family members in the global population. Results are calculated according to Kimura and Ohta [65] and Wright [56] under the neutral process (see the formula in main text). Parameters used in the figure are the number of local populations *n*=50, the effective size of local population $N_{e\left(\mathrm{local}\right)}$=50, the mutation rate per family member per generation *v* = 10^-5^ , the constant *a*=0.1, the rate of interchromosomal crossing-over per generation *β* = 0.001. *Y*-axis represents the identity coefficient between family members with the recombination rate (distance) *x*=0.1 on a chromosome. Note that migration within the global population does not change other parameters in *f*(*x*) except the effective population size.

In theory, population differentiation can affect the correlation blocks (gametic or zygotic LD) in the global population in terms of family member. Fig. (**7**) shows how population structure (*F_st_*) changes the identity coefficient between the gene family members, based on the synthesis of the results by Wright [56] and Kimura and Ohta [65]. Results are calculated by substituting *N* in *b* (=2*Nβ*/(1+4*Nv*)) of Kimura and Ohta’s Eq. (18), the identity coefficient between family members with the recombination rate,

fx = e−abx2abx∫0∞e−2abxtt1+ta/b/2dt, with Neglobal = nNelocal/1−Fst

 under the neutral process [56]. *a* is the constant related to intrachromosomal unequal crossing over. Population differentiation (*F_st_* ≠ 0) increases the effective global population size and hence facilitates the inter-chromosomal crossing-over, which in turn reduces the genetic correlation (Fig. **7**). This result implies that local population differentiation facilitates the divergence in the correlation block size in the global population.

Analogous to its effects on population differentiation, genetic drift aids to diversify the pattern of *F_st_* -correlation block in terms of multigene family. Populations with small effective sizes increase the fixation probability of the maladaptive variants [68, 69] and impede the spread of the adaptive variants to all the family members through unequal crossing-over or gene conversion. This is in contrast to the outcome in populations with large effective sizes.

Current challenge is to decipher the relative contributions of different evolutionary forces in maintaining the multigene family [70, 71]. It is necessary to develop methods that evaluate the observed pattern of correlation blocks in terms of multigene family to better understand the underlying evolutionary processes. This is feasible for species whose family members can be mapped from their whole genome sequences to enable to the analyses of their correlation blocks.

### Perturbation from Transposable Elements

The processes for maintaining the number of TE copies in a population are complex [72-76]. The effects of transposition on the host genomes are associated with the intensities of selection on (i) the transposable elements themselves (positive or negative) and (ii) the modified host sequences. A positive effect facilitates the spread of TE in a population until other forces such as genetic drift counteract their replication [77]. The number of TE copies does not increase infinitely although the number of potential sites for transposition is sufficiently large [73, 76]. When negative effects are acting on the host genomes, such as insertion into the coding regions, the abundance of TE is maintained by the balance between selection and replication [72, 73]. When the selection intensity is of the order similar to the effect of genetic drift, the mechanism of replication-drift cannot be excluded.

Empirical studies demonstrate that TE can be sources of variation via its insertion into different regions of a gene, such as in exons, introns, and regulatory regions of host genes (see review by Kidwell and Lisch [74]). The perturbation from TE on the correlation blocks within subpopulations is likely related to how and where the transposition has occurred on the host genomes. When neutral TE are inserted into the non-coding regions that are adjacent to the selective sites [74], the original correlation blocks likely expand or become more separated due to the extension of neutral segments and the effects of genetic hitchhiking. In contrast, when neutral TE are inserted into the adaptive coding regions [74], the original correlation blocks likely break into smaller blocks and their number increases. When selective TE are inserted into the non-coding regions, new blocks likely arise and their block sizes are related to the strength of selection against the TE due to genetic hitchhiking effects [78]. When selective TE are inserted into the coding regions, the original block sizes could change to various degrees and this probably depends on how far the TE are located away from the original selective outliers. These conjectures suggest that a complex relation might exist between the effects of TE and the pattern of correlation blocks. 

Similarly, a complex relationship might exist between the effects of TE and *F_st_* -correlation blocks. Studies have shown population differentiation for TE under genetic drift, mutation and other forces [79-81]. The differential selection against the same TE facilitates unequal TE abundances among the populations. In addition, the difference in the effective population sizes enhances to generate unequal genetic drift effects on the spread of TE among the individuals. Like the existence of finite number of TE in a subpopulation, the joint effects of multiple forces (e.g., selection and genetic drift) on the spread of TEs eventually will lead to a finite number of *F_st_* -correlation blocks.

Migration and demography history can affect the dynamics of TE within the genomes and structured populations [81]. The distribution of TE copy number among subpopulations can be modified by the relative migration rate, transposition rate, and the strength of selection against the deleterious effects of TE. The homogenization process for the TE copy number due to migration may likely take a long time in structured populations. Similarly, inter-population migration homogenizes the perturbation effects on *F_st_* -correlation blocks. One likely consequence is that migrating genomes could change different TE copy numbers and hence the number of blocks in the recipient populations when TE are neutral or nearly neutral under the infinite-allele model. This is analogous to the increase in the rare allele richness (or rare species richness) due to the effects of immigration under the infinite-allele model (or infinite-species model) in molecular population genetics (or in neutral community ecology) [76, 82-84]. The other likely scenario is that immigrating TE can cause migration loads when the migrating TE are maladaptive in the recipient populations. This consequently alters the pattern of F_st_ -correlation blocks. The above analyses suggest that a very complex pattern of F_st _-correlation blocks might occur under the joint effects of migration with other forces. 

**Fig. (8):**
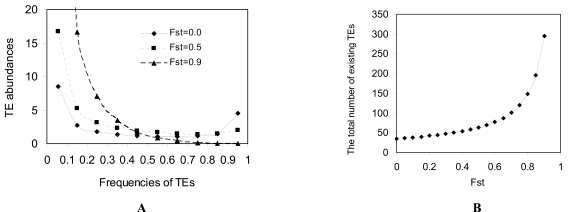
Effects of population differentiation (*F_st_*) on the distribution of transposable elements (TEs) in the global population: **A**. TE abundances under different frequencies; and **B**. Changes of the total number of existing TEs with *F_st_* . Results are calculated according to Ohta [76] and Wright [56] under the neutral process (see the formula in the main text). In Figure A, the values on X-axis represent the intermediate values of fixed frequency intervals: [0.01, 0.1], [0.1, 0.2], …, and [0.9, 1.0]. Y-axis represents the estimated TE abundances corresponding to the fixed frequency intervals $\left(=\int_{\mathrm{x1}}^{\mathrm{x2}}G\left(x\right)\mathrm{dx}\right)$. In Figure B, the total number of existing TEs is estimated by $\int_{1/2N_{e\left(\mathrm{global}\right)}}^{1}G\left(x\right)\mathrm{dx}$. The common parameters used in both figures are the number of local populations *n*=30, the effective size of local population $N_{e\left(\mathrm{local}\right)}$=30, the transposition rate *v* = 0.0001, and the average number of TEs per genome *n_TE_* =10.

Population differentiation can affect the distribution of TE abundance in the global population, and this subsequently affects the correlation blocks within and between subpopulations. Population differentiation can increase the number of transposable sites for those TE with low frequencies in the global population under the neutral process (Fig. **8A**). These were calculated by substituting N in $\theta \left(={4N}_{e\left(\mathrm{global}\right)}v\right)$ of Eq. (2) of Ohta [76], 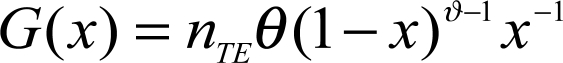, by $N_{e\left(\mathrm{global}\right)}={\mathrm{nN}}_{e\left(\mathrm{local}\right)}/\left(1-F_{\mathrm{st}}\right)$ under the neutral process [56]. G(x) is the function so that *G*(*x*)*dx* represents the number of TE transposable sites whose frequencies are within x ~ x+dx and the sum of the allelic frequencies is 1. Large population differentiation increases the effects of those TE with low frequencies on the correlation blocks in the global population. Population differentiation also facilitates the accumulation of the total number of existing TE (Fig. **8B**). However, this can be modified in the non-neutral process where the effective size of the global population reduces due to the variation in fitness among populations [57, 72, 73].

Mutation could lead to changes in the structure of TE, and hence affects its function on the host genomes, as implied from studies on the type of TE and their evolutionary relationships [70, 85]. The consensus is that favorable TE mutants would facilitate their spread in population which otherwise could be rapidly removed from their resident populations. The fate of new TE mutants (extinction or persistence in sub-/ global-population) could influence the correlation blocks and this awaits further research. 

The effects of TE perturbation further complicate the assessment of the correlation blocks within and among populations and their distribution. One probable way is to check the TE from the genome sequences of model organisms and to investigate their diversities within and among populations [45]. This helps to predict whether the perturbation of TE is negligible in modifying the number and sizes of correlation blocks. 

In general, perturbation of TE increases uncertainty in the size of correlation blocks, leading to a dynamic distribution in block number and size. Whether the effects of such perturbation are linearly additive remains to be studied, but this uncertainty could likely be substantial, partially depending on the function of TE, their abundances and effects on host genomes.

### Effects of Nongenic Sequences and GC Isochores 

The genomic structure of eukaryote is characterized by abundant repetitive inter-dispersed nongenic sequences, such as in the pine genomes [86]. The highly repetitive sequences each with a few to hundreds of nucleotides aid in the formation of correlation blocks within populations, especially when the highly repetitive sequences are neutral and act as the inter-spacers flanking correlation blocks. The repetitive sequences with hundreds to thousands of nucleotides facilitate the formation of correlation blocks of middle sizes when they are selective and contain outliers, which otherwise functions as the highly repetitive sequences. Tandem repetitive sequences are expected to be less effective than the interspersed repetitive sequences in shaping the number and size of correlation blocks within populations. The single-copy sequences often code functional genes and contain outliers, such as *H_e_* and *F_st_* outliers. Empirical studies are unavailable to examine the relations between repetitive sequences and correlation blocks.

The processes that maintain repetitive sequences (mainly the nongenic DNA) are complex. These include transposition, replication slippage, unequal sister-chromatid exchange and inter-chromosomal unequal crossing-over [70, 87]. Some of these have been discussed in the preceding two subsections. The process through recombination within and between chromosomes is affected by the recombination heterogeneity along the chromosomes [29, 31]. As well, the spread of tandem and interspersed repetitive sequences can be mediated through different paths in a population. For instance, variation in the number of tandem repeats (VNTR) among the chromosomes implies high polymorphism among the individuals within populations. However, the number of repetitive sequences should be finite owing to the balance between extinction by genetic drift and the formation by replication (one kind of mutation), provided that the repetitive sequences are neutral. The distribution in the number and size of repetitive sequences among the individuals varies with populations of different effective sizes [88], facilitating the formation of distinct *F_st_* -correlation blocks. 

Migration reduces population difference in the number and size of repetitive sequences, given that migrating genomes recombine with the genomes in the recipient populations. The presence of nongenic repeats increases the probability of occurrence of genetic hitchhiking [89], and hence modifies the *F_st_* -correlation blocks. However, this condition infrequently occurs in the prokaryotic genomes where nongenic DNA is absent or accounts for a very small proportion of the genomes [70].

Another constitutional feature comes from the presence of GC-rich isochores that form a mosaic pattern within chromosomes and related to the recombination hotspots [29, 90, 91]. Complementary to the tandem and interspersed repetitive sequences that are mainly nongenic, GC-rich isochores are mainly distributed in the coding regions although the mechanisms for their originations is still in dispute between selectionists and mutationalists [70]. The pattern of correlation blocks within and between populations in terms of GC-rich isochores is expected to exist from the point of either selectionists’ or mutationalists’ view. Different natural selection intensities among GC-rich isochores can result in correlation blocks of various sizes due to genetic hitchhiking effects, as implied from human genome studies [89]. The distribution of correlation blocks may be diverse from those in terms of other units (e.g., TEs or multigene families). Mutational differential among GC-rich isochores can reinforce a mosaic pattern of genomic diversity. Difference in effective population sizes or in selection intensities can result in a mosaic distribution of *F_st_* -correlation blocks in terms of GC-rich isochores while migration tends to homogenize these differences.

### Perspectives

When distinct assembly features as multigene families, TE, and repeats are jointly considered, the challenge is how to distinguish each from the observed pattern of the correlation blocks, or how to assess their relative contributions to this pattern. The preceding discussions suggest the complexity of the processes that maintain their dynamics. These are briefly summarized in Table **1**. The relative contributions of different attributes differ among species. For example, the non-genic repeats probably play a more important role in pines but not in the prokaryotes since pine genomes contain a substantial amount of nongenic repeats [86]. The effects of TE perturbation are likely important in the genomes of human and other mammals since a majority of their repeats are TE [17]. For a given species, one intuitive approach to evaluate their relative contributions is to compare the number and sizes of the correlation blocks by partitioning the total variation into the different process components and testing for their significance. The challenge of such an analysis is to identify the individual blocks in the presence of diverse evolutionary processes.

## CONCLUDING REMARKS

Correlation blocks and their distribution along the chromosomes are an important aspect of the structure of genomic diversity at the population level. Study on genomic structure requires data on genome-wide SNPs or markers that not until recently are available in a genetic studies of population structure. The present synthetic review attempts to tie population structure with genomic structure by bring forth their complex interfaces. Our discussions address how population structure shapes the pattern of correlation blocks and how the evolutionary processes affect the pattern of correlation blocks. Methods for characterizing the pattern of correlation block, such as the correlation of *H_e_* ’s (genomic diversity structure within subpopulations) and the correlation of *F_st_* ’s (genomic diversity structure among subpopulations), have been presented. 

The consensus is that correlation blocks of various sizes do exist, and their numbers and sizes will diminish as SNP maps become progressively denser as in the case of haplotype block size in human genomes. With the availability of population genomic data in many species, it has become increasing important to quantify and characterize the amount, distribution and pattern of correlation block at the population level. This provides a population-based genome-wide perspective when developing strategies in conservation biology, given that the number of correlation blocks is analogous to the effective number of “super sites” (removing the redundant information from correlated diversities within each block). In the eukaryotic genomes, the distribution pattern of correlation blocks is associated with the genomic assembly features. Multigene family, non-genic repetitive sequences and GC-rich isochores may reinforce the pattern of correlation blocks. Perturbation from transposable elements increases the uncertainty of this distribution pattern in size and number of correlation blocks. There is a considerable opportunity to explore and elucidate the relationships between the structure of genomic diversity and the evolutionary processes. 

## Figures and Tables

**Fig. (2):**
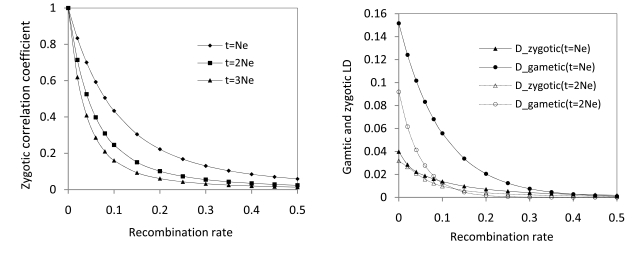
The change for zygotic and gametic LDs between linked neutral sites. **A**. Correlation of heterozygosities decays with the time measured in terms of effective population size (*N*_e_) and with the distance measured in terms of recombination rate. **B**. A comparison between the heterozygosity disequilibrium and the gametic linkage disequilibrium. The result indicates that the gametic LD decays faster than the zygotic LD within short distances although the gametic LD is greater than the zygotic LD in magnitude. Calculations are based on synthetic theories, Eqs (1) ~ (3). The initial settings are *N*_e_ =10, the gametic linkage disequilibrium=0.25, and the allele frequency at each of two diallelic sites=0.5.

**Fig. (5):**
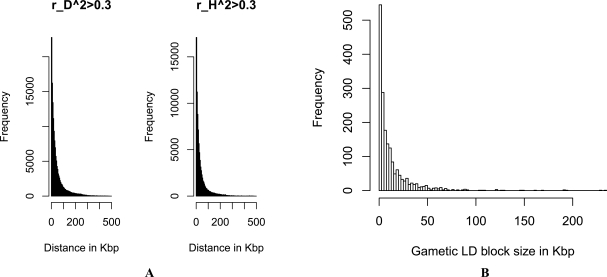
**A**. Distribution of pairwise correlations of gametic ( $r_{D}^{2}$ >0.3) and zygotic ( $r_{H}^{2}$ >0.3) LDs with distance, indicating a negative exponential distribution on human chromosome 21 in CHB (Han Chinese Beijing) population. There were 145801 pairwise gametic LDs with $r_{D}^{2}$ >0.3 and 87411 zygotic LDs with $r_{H}^{2}$ >0.3 and the bin size was set as 5Kbp. **B**. The abundance distribution of gametic LD sizes with distance on human chromosome 21 in CHB population. Gametic LDs were measured by Lewontin’s *D*’ and results are obtained using HaploView [1]. There were 1811 gametic LD blocks ( *D*’ ~1.0) and the bin size was set as 2.5kbp.

**Fig. (6):**
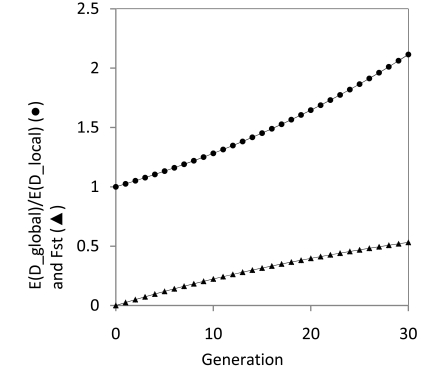
A comparison between the global and local LDs in a pure genetic drift process. The results indicate that the expected global LD at generation *t*, $E\left(D_{\mathrm{global}\left(t\right)}\right)$ , is greater than the expected local LD, $E\left(D_{\mathrm{local}\left(t\right)}\right)$ . Results are calculated based on Eqs. (8) ~ (12), with the effective size of each subpopulation $N_{e\left(\mathrm{local}\right)}$ = 20 and the number of subpopulations *n*=50.

**Table 1:** The Evolutionary Processes and their Potential Effects on Correlation Blocks within and Among Populations

	Selection	Mutation	Migration	Drift
Multigene Family	Facilitating the homogeneity between family members within populations.	Selective mutation within family members may change the pattern of correlation blocks.	Homogenizing the structure of genomic diversity between populations.	Enhancing the differential structure of genomic diversity among populations.
	Different selection strengthes between populations change *F_st_* -correlation blocks.	Neutral mutation has no effects.		Enhancing the heterogeneity between family members.
Transposable elements (TEs)	Insertion of selective TEs may change the original correlation block size	Changing the structure and function of TEs, and hence the pattern of correlation blocks.	Homogenizing TEs effects among populations.	Affecting the spread of TEs on host genomes
	Insertion of neutral TEs into coding regions may change original block size.		Migration of maladaptive TEs produces migration load.	Enhancing the differences in diversity of genomic structures among populations
	Insertion of neutral TEs into noncoding regions may expand original correlation block size.		Migration of neutral TEs enhances the number of small correlation blocks to the recipient population.	
	Differential selection strengthes among populations may change *F_st_*-correlation blocks.			
Repetitive nongenic sequences, GC-isochores	Enhancing the probability genetic hitchhiking effects.	GC-isochore mutation enhances a mosaic pattern of correlation blocks.	Reducing the number and size of repetitive sequences.	The abundance of repeats controlled by replication and drift.
	Distinct selection strengthes among GC-isochores enhance different patterns of correlation blocks.		Homogenizing the different patterns produced by GC-isochores among populations.	Different *N_e_*’s enhance different patterns of correlation blocks in terms of GC-isochores
	Distinct selection strengthes on GC-isochores among populations change *F_st_*-correlation blocks.			
